# The Use of Compressed Air for Desensitizing Sensitive Dentin

**Published:** 1913-09-15

**Authors:** W. T. Reeves

**Affiliations:** Chicago


### THE USE OF COMPRESSED AIR FOR DESENSITIZING SENSITIVE DENTIN.

BY W. T. REEVES, D.D.S., CHICAGO.

In giving to you tonight this short paper on the use of compressed air for obtunding sensitive dentin, I believe I am bringing you a very simple and effective means to that end. In presenting this subject we have two conditions to contend with—physical and mental. We will first take up the physical. The production of cold artificially is affected by various means. When a body, in the broad sense, is rarefied, passing from the solid to the liquid or the gaseous, or from the liquid to the gaseous state, or, being a gas, expands against pressure energy is generated. This energy is practically supplied as heat. If none is directly applied, and the rarefaction is produced without heat then the body expands its own heat energy in the mechanical or physical work of rarefaction and cold is produced. The volatilization of liquids is one of the best known means of producing cold and in dentistry we use ether, and ethyl chlorid to that end, but the intense cold produced when applied to a tooth is often more painful than the drilling and excavating would have been. The phenomenon of cold produced by rarefaction of a gas is well illustrated by a cylinder of nitrous oxid gas. Here we have a hundred gallons of gas compressed into a receptacle that would hold about a quart of liquid. The volume of compression or pounds pressure I don't know, but if liberated in the right quantity, rarefaction or its return to orignal volume will produce cold intense enough to form frost and ice from the moisture in the atmosphere, sufficient to close the orifice. Air can be compressed in volume until it becomes a liquid. The compression of air or any other gas produces friction and friction means heat. But when at rest the tank and contents will soon radiate its heat until it is practically the same temperature as the surrounding atmosphere. The greater the compression the greater the cold produced in rarefaction. According to volume of compression, or pounds pressure, so must be the size of the orifice of escapement that energy produced by rarefaction may result in cold. In speaking of compression I shall use the term pounds pressure as conveying a more comprehensive understanding than the term atmosphere. If we had a tank containing air at 20 pounds pressure and allowed it to escape through an orifice half inch in diameter very little energy would be engendered by its rarefaction and consequently very little cold produced, but if on the other hand the orifice was the size of a needle point, the energy produced by rarefaction would produce quite a satisfactory degree of coldness.

From the foregoing you will see that the distance from which you deliver the air upon the object will have everything to do with the degree of cold produced. You can be so far away from the object that rarefaction has reached the stage of full expansion, or so close that the jet of air is shunted to one side before rarefaction starts, and in either case no results are obtained. Compressed air at 20 lbs. pressure will produce fair results for obtunding sensitive dentin; 30 lbs. pressure will do better; but 40 lbs. pressure is desirable—50 lbs. or more will give still better results. I have taken 40 lbs. pressure as desirable, because that is the average pounds pressure of the compressed air furnished in the different office buildings in the city and have worked out my deductions on the basis of 40 lbs. pressure. Compressed air at 40 lbs. pressure delivered in a jet from an orifice the size of a No. ½ round bur into a cavity such as we deal with in cavities of decay in a tooth at a distance of from a quarter to a half inch, will reduce the object to a temperature of about 52 degrees. Body temperature is 98 degrees Fahrenheit—freezing point is 32; a difference of 66 degrees. Reducing the tooth to 52 degrees, we have reduced the temperature of the tooth 46 degrees, or a little over two thirds from body heat to freezing point. As a matter of fact if the temperature of the body is reduced to about 50

degrees the body is so numb from cold as to have very little feeling or sensation. That is what we do when we apply compressed air at 40 lbs. pressure to a cavity of decay in a tooth. The tooth is lowered to a temperature of about 52 degrees and the pulp is so benumbed as to respond not at all, or very feebly to the pain sensation produced in the use of bur, stone or excavator in cavity preparation. Secondly, and of no less importance, we absolutely prevent all heat from friction in the use of bur, stone or excavator. Heat from friction is the main cause of pain sensation to the brain in cavity preparation.

Unfortunately we have another condition to contend with—the mental or psychological effect of work upon the teeth. The brain receives impressions through the five senses: taste, smell, sight, hearing and touch; so far we have only dealt with touch. Sight and hearing play an important part in the conveyance of pain sensation to the brain, hearing being greater than the others. The brain, through repeated telegraphic communications from the five senses has become so used to receiving certain sensations under certain conditions, that such sensations are automatically recorded; that is, the brain receives and records automatically a sensation quicker than the brain can think and reason that such a condition has transpired.

Unfortunately by the great majority of people the dental chair is looked upon as a chair of torture, and to such pain sensations are automatically recorded through any of the five senses; namely sight, hearing and touch. The only exception is a child who has not experienced any previous dental operations or has not experienced pain from toothache, or to whom the parents has not held up the dentist as a bogy man. Such a patient I have no trouble to work for—never deceiving them, letting them know they feel this or that, when you know feeling will result from the part of the cavity you are working in. They not being scared, will receive no greater pain sensation than from the same amount of feeling in any other part of the body.

Sight performs a considerable part in the automatic registration of pain sensations in dental operations. How many of you have seen patients cringe and experience pain at the approach of an object of dental operation toward the mouth. That is pain sensation automatically registered before reason can act and convince the patient that such condition has not existed. The pain is just as real even if the registration has been false instead of true. Hearing plays a greater part in false registration than sight, and unfortunately for us, we can hear through the teeth about as well as through the ears. It is the noise from the jump of the bur in the cavity or the click of the bur upon the enamel that automatically records a false impression of pain. You have all had the experience of working upon a devitalized tooth and the patient experiencing all the pain sensation that would come from a vital tooth. That is the false registration of pain sensation through the sense of hearing. Another illustration of false registration of sensation; at some time or other I expect you have all had the experience of approaching a radiator that you expected was hot, and upon touching it a burning or heat sensation has been conveyed by the extreme cold before reason could act and convince you to the contrary.

Now for this condition you have got to produce a psychological effect—for the majority of patients it will not answer to simply obtund the sensation of touch. You have got to produce a psychological effect. That is, you have got to prepare the mind to receive the benefit that comes from the obtunding of the sensation of touch. The application of compressed air through the ordinary air nozzle used as a chip blower would to the majority of patients convey only pain sensation, for that is what they have usually experienced from the chip blower, and all your logic could not convince them to the contrary.

For the psychological effect upon the patient I employ an ordinary spray bottle in which is placed a vial containing chloretone. I tell them that chloretone is a synthetic drug produced to take the place of cocain, but that its effect upon dentin was not profound enough—until compressed air was employed to force it into the dentin. You are telling them no untruth, but you have prepared their mind to receive the benefit that comes from the obtunding of the sensation of touch.

To summon up results, I believe I am very conservative when I state that fully 75 per cent. of all cavities for all conditions of patients are prepared without any pain sensation and of the other 25 per cent. of cases I have from 75 to 90 odd per cent. of success. This method is so simple as to be almost unbelievable, but if you will make a rational application of this method I believe any and all can obtain these results. Bear in mind all the time that you are working upon a vital tooth, and work accordingly. You could force the cutting so hard that nothing short of complete anesthesia could prevent pain sensation.

The modus operandi—no previous preparation of the patient is necessary. You operate without putting on the rubber dam. The saliva syphon is placed and cotton rolls where needed, and changed when necessary. Your assistant now starts the application of the air, beginning with a jet so minute that it is not felt by the tooth. If it should be felt, place a pledget of cotton into the cavity—and direct the jet of air upon that. In a few seconds the tooth will become used to the air and the cotton removed. Gradually increase the air until you are giving the full force of the 40 lbs. pressure, taking about a minute and a half. In the meantime you will lay out on the table what instruments, burs and stones you expect to use in preparing the cavity. Your assistant keeps the air playing into the cavity all the time without any let up—if the air was stopped sensitiveness would return to the tooth in about a half minute. Now begin on a part of the cavity that you know will not hurt, and in that way let the fact that you are cutting the tooth and that it is not hurting sink into the mind of the patient. Soon you will go into other parts of the cavity and have it all completed before you know it, and the patient will not have suffered any pain—and lastly, the whole operation is so simple and quickly performed that you would be all through while other methods were getting their paraphernalia adjusted.

